# Changing trends of blindness, visual impairment and cataract surgery in Bhutan: 2009–2018

**DOI:** 10.1371/journal.pone.0216398

**Published:** 2019-05-09

**Authors:** Nor Tshering Lepcha, Indra Prasad Sharma, Yuddha Dhoj Sapkota, Taraprasad Das, Tshering Phuntsho, Ngawang Tenzin, Bindiganavale Ramaswamy Shamanna, Sonam Peldon

**Affiliations:** 1 Department of Ophthalmology, JDW National Referral Hospital, Thimphu, Bhutan; 2 International Agency for Prevention of Blindness, South East Asia Regional Office, Banjara Hills, Hyderabad, India; 3 International Agency for Prevention of Blindness, South East Asia Region, Kallam Anji Reddy Campus, L V Prasad Marg, Hyderabad, India; 4 School of Medical Sciences, University of Hyderabad, Hyderabad, India; 5 Primary Eye Care Program, Ministry of Health, Thimphu, Bhutan; Universidad de Monterrey Division de Ciencias de la Salud, MEXICO

## Abstract

**Purpose:**

To obtain new rapid assessment of avoidable blindness (RAAB) data on the prevalence, causes and trends of blindness, visual impairment and cataract surgery; and compare the new 2018 data with the older RAAB 2009 data.

**Methodology:**

The second nationwide RAAB used android based mRAAB technique and technology for data collection. Using the compact segment sampling probability proportionate to size, 5,050 participants from 101 clusters of 50 people aged 50 years and older were enrolled through house-to-house visits. Eligible participants received ophthalmic examination and interview by the ophthalmologist-led emuneration team as per the RAAB protocol.

**Results:**

The age-sex adjusted magnitude of bilateral blindness in Bhutan was 1.0% (95% Confidence Interval, CI 0.5–1.4) with relatively higher prevalence in rural population (Odds Ratio, OR 1.5, p = 0.13) and women (OR 1.6, p = 0.06). Untreated cataract accounted for the most of blindness (53.8%), severe vision impairment (57.1%), and moderate visual impairment (65.3%); uncorrected refractive error was the main cause of early visual impairment (46.7%). Cataract Surgical Coverage was 86.1% with relatively better coverage in men (76.7% men; 73.1% female) and urban population (79.2% urban; 70.2% rural). Good cataract surgical outcome was achieved in 67.3% and leading cause of poor outcome was ocular comorbidity (43.6%). Accessibility was a significant barrier to the uptake of cataract surgical services.

**Conclusion:**

There is a 33% reduction in blindness from 1.5% to 1.0%, since the first RAAB survey in 2009. In order to further reduce blindness and visual impairment, Bhutan should continue to implement long-term strategic action plan for eye health focused on strengthening primary eye care and comprehensive eye care service.

## Introduction

Blindness and visual impairment (VI) are major public health challenges. Universal Eye Health: a Global Action Plan (GAP) 2014–2019, adopted by the World Health Assembly 2013 (WHA 66.4) called for action plans to 25% reduction of blindness and visual impairment by 2019 from its baseline of 2010. [1] The plan recommended the member states to conduct population based surveys to provide evidence on visual impairment and its causes for planning and evaluating eye health programmes. The Rapid Assessment of Avoidable Blindness (RAAB) is a rapid and reliable and accepted method to estimate the prevalence of blindness and visual impairment in the population over 50 years. [2] Assessing the elderly population, 50 years or older, is cost effective because majority of blindness (82%) and moderate to severe visual impairment (MSVI, 65%) occurs in this age group. [3, 4]

Bhutan provides free access to basic public health services to its citizens as its constitutional mandate and the primary eye care program is integrated into the primary health care system. [5] In Bhutan, the first RAAB survey was conducted in 2009; it documented 1.5% blindness in people ≥ 50 years age. [6] The results of the first RAAB were used in program planning to improve the eye care services in the country, including investment in human resource, infrastructure, primary eye care and outreach programs. Further, the Primary Eye Care Program (PECP) at the Ministry of Health was entrusted with a greater responsibility on the eye health activities in the country.

This second follow-up nation wide RAAB survey was conducted approximately 8 years after the first one, and was in alignment with the mandates of the WHO-GAP 2014–2019. It was planned and executed to generate evidence on the present magnitude and causes of blindness and vision impairment, evaluate the status of the cataract services, and assess the impact of the existing eye care programme in Bhutan. The result of the survey is expected to provide the trend of blindness in the country that could be used to further reinforce programmatic initiatives for specific long-term strategic action plan for eye health services in Bhutan.

## Methodology

This cross-sectional population based study was conducted using the standardized RAAB methodology between December 2017 and February 2018. It was executed by the PECP, Ministry of Health with technical support from the International Agency for Prevention of Blindness (IAPB), South East Asia region and funding support of the Lions Club International Foundation (LCIF).

### Study participants

The study enrolled, examined and interviewed all individuals aged 50 years and older, residing in the household for six months or more over the past year in the selected study cluster. Individuals who were unable to communicate or refused to participate were excluded.

### Sample size calculation

The population of Bhutan was 779,666 and the target population aged over 50 years was 115,178, according to the population projection of the National Statistical Bureau of Bhutan (NSB). [7] The expected prevalence of bilateral blindness (best corrected visual acuity, BCVA< 3/60 in the better eye) was 3% (among age 50 years and above) in the South East Asian region. [8] The sample size and study clusters were selected using RAAB 6 computerized software. With a confidence interval of 95%, a relative precision of 20%, design effect (DEFF) of 1.5 and a non-response rate of 10%, the calculated sample size was 5,040. Considering the cluster size of 50 people aged 50 years and older, a total of 101 clusters was selected using the utility of random selection in the RAAB software.

### Sampling method

The survey sampling frame representing all 20 districts was created based on the population data of the NSB, which divided the rural and urban population into 1,044 and 1,529 population units respectively. The rural to urban population in Bhutan was 70:30, hence, 70 rural and 31 urban clusters were selected by systematic sampling with a probability proportionate to size using RAAB6 software. [9] Each cluster had a population ranging between 250–500 so as to give at least 50 people ≥ 50 years of age in each cluster.

The country was divided into three regions and detailed maps of selected study clusters were obtained and prepared. One survey team was assigned to each region. Households were selected through compact segment sampling. After starting from a randomly selected household, other households were chosen sequentially in the direction chosen until 50 subjects aged 50 years or above were enrolled. If the segment did not include enough eligible participants, the survey was continued in another segment that was randomly chosen and the process continued until a cluster size of 50 individuals was achieved. In cases where the team came across a locked house, it was checked with the neighbors if any eligible person was living there and enrolled accordingly; 2 attempts were made to examine them before deciding that they were not available.

### Training and quality assurance

Three survey teams were formed comprising of 4 members including an ophthalmologist who led the team. One of the teams had an optometrist (IPS) in lieu of ophthalmic assistant who also actively participated in the study design and planning. The teams received comprehensive training prior to the fieldwork by certified RAAB trainers (YDS, BRS) for 5 days to ensure data quality and strict adherence to the study protocol. It included a discussion on the design of RAAB, data collection procedures using mRAAB, clinical examination procedures, inter-observer variation (IOV) assessment and a pilot study.

IOV was tested between all teams using repeat examination of 50 participants to ensure high degree of consistency between the teams. The most experienced team led by the Principal Investigator (NTL) was considered the ‘Gold standard’ team. The examaminations included measurement of visual acuity, evaluation of the lens and assigning the cause of visual impairment. During the IOV, the teams obtained acceptable agreement with a minimum kappa score of 0.67 for all parameters. The training program also included a pilot study in one of the non-selected clusters with the trainers. Data analysis of the pilot cluster was done to compare the findings of each team and group discussion was conducted to minimise the inconsistencies. After the training, the national RAAB trainer accompanied each team in turn for a period of one week each during the study period to check the reliability and validity of data collection.

### Ophthalmic examination and data collection

The standard RAAB Protocol (Version 6.0) was used for ophthalmic examination. [10]

Visual acuity (VA) was measured with available correction in daylight illumination for each eye using the Snellen Tumbling ‘E’ letter with optotype sizes 6/12, 6/18 and 6/60 at 6 m, 3 m and 1 m. If the VA with available correction was *<*6/12 in either eye, then the pinhole vision was measured following the same procedure.

The distant direct ophthalmoscope was used in a shaded or semi-dark area without pupillary dilatation for assessment of lens status. Dilated (1% tropicamide) fundus examination was performed by the ophthalmologist to assign the principal cause of blindness or VI when the pinhole VA was *<*6/18 and and there were no obvious media opacities due to cataract or corneal opacity. One cause that was responsible for vision loss in the person or the eye was considered the principal cause of the blindness or VI. The surgery history and related information were collected from the people earlier operated for cataract.

All participants received feedback about their eyes and were advised to seek ophthalmic consultation if they had any concerns. Appropriate counseling and referral to the nearest regional or national referral hospitals were done after the examination.

### Ethics approval

The study conformed to the tenets of the Declaration of Helsinki. The protocol was reviewed and approval granted by the Research and Ethics Board of Health (REBH), Ministry of Health, Bhutan (Ref. No. REBH/Approval/2017/075); administrative approval was obtained from all relevant agencies. Prior to the enrollment and examination, all eligible participants were explained the study procedures in their local dialect and the attendant benefits such as to identifiy the cause of blindness/ visual impairmant and help people reach the treatment center, if needed. Written informed consent with signature or inked fingerprint (specially from blind and/or illiterate people) was obtained stating their acceptance to voluntary participate in the study and permit use of the data for publication. The procedure was explained verbally to bilaerally blind people in presence of their relative/friend before inked fingerprint was obtained. No financial incentives were provided for participation in the study.

### Definitions

Standard definitions were used to define the cataract surgical rate (CSC) and effective CSC (eCSC) as follows: CSC- proportion of patients (or eyes) with ‘operable’ cataract, who have already received surgery. [11] eCSC- combines the coverage measure, CSC with quality (postoperative visual acuity > 6/18). [12] Visual impairment (VI) and blindness were categorized by the measured visual acuity (VA) as follows: blind- VA < 3/60; Severe visual impairment (SVI)- VA <6/60–3/60; moderate visual impairment (MVI)- < 6/18–6/60; and early visual impairment (EVI)- <6/12–6.18. [13]

### Statistical analysis

The software program (RAAB Version 6) which has an inbuilt standardized format for data analysis and display of results was used. The data collected by each team were sent via application supported emails to three survey coordinators after completion of each cluster for backup data bank. Consistency checks were performed by the coordinators based at the center and inconsistencies were adjusted on the same day.

The prevalence estimates took account of the DEFF (design effect) while estimating the confidence intervals. The results were recorded by person (bilateral blindness or VI) and by eye (unilateral blindness or VI). Age and gender adjusted prevalence were calculated with reference to the national estimates. The visual outcome of cataract surgery was reported among all the pseudophakic or aphakic eyes. The CSC was calculated by eye and person. Since the presenting visual acuity (PVA) prior to the surgery was not known, assumptions were made that only patients with visual acuity below a threshold of <6/18 underwent cataract surgery.

## Results

### Study population

The three survey teams enrolled 5,050 people from 101 clusters representing 4.4% of all people aged 50 and above from all 20 districts. Among the enrolled participants, 0.6% (n = 28) people were not available for examination, 0.9% (n = 44) people were physically or mentally not able to communicate and 0.25% (n = 8) people refused examination. Consequently, 4,970 persons (response rate 98.4%) which included 48.2% (n = 2,398) men and 51.8% (n = 2,572) women, underwent eye examination. As per the information obtained from the neighbors, no one was blind among the non-participants, but in absence of measured visual acuity, we do not know their visual impairment, if any. The recruited study population constituted 69.3% rural and 30.7% urban with similar response rates (98.3% vs 98.7% respectively). The sample population differed from census population with more older people represented in the study compared to younger age groups of 50 to 59 years old S1 Table, hence, these differences were adjusted in the results and presented as age-gender adjusted results to estimate the prevalence.

### Prevalence of blindness and visual impairment

The age and gender adjusted prevalence of bilateral blindness, severe visual impairment, moderate visual impairment and early visual impairment with available correction for population aged 50 years and older in 2018 was 1.0% (95% Confidence Interval, CI, 0.5–1.4), 0.6% (95% CI, 0.4–0.9), 5.0% (95% CI, 4.2–5.8), and 7.6% (95% CI, 6.6–8.5), respectively. The prevalence of blindness was higher in women (1.3% women and 0.6% men; OR 1.5, p = 0.13) and in rural area (0.9% rural and 0.7% urban; OR 1.6, p = 0.06) though these differences were statistically not significant. The prevalence increased with aging; 0.4% in the age group of 50 to 59 to 6.5% in age group of 80+ years. The changes in the prevalences of blindness, SVI and MVI between the first RAAB (2009) and current RAAB (2018) is shown in **Table 1**.

**Table 1:** Prevalence of blindness, SVI, MVI and EVI; 2009 and 2018.

Vision Category	2009 % (95% CI)			2018 % (95% CI)			Changes in %
	Male	Female	Total	Male	Female	Total	
**Blindness, PVA<3/60**	1.4 (0.9–1.9)	1.6 (1.1–2.2)	1.5 (1.1–1.9)	0.6 (0.2 to 1.1)	1.3 (0.6 to 2.0)	1.0 (0.5 to 1.4)	33% decrease
**SVI, PVA** **<6/60 to 3/60**	0.9 (0.4–1.4)	1.5 (0.9–2.0)	1.2 (0.8–1.6)	0.7 (0.3 to 1.1)	0.5 (0.2 to 0.9)	0.6 (0.4 to 0.9)	50% decrease
**MVI, PVA** **<6/18 to 6/60**	4.4 (4.04–5.75)	5.6 (4.4–6.7)	4.9 (3.9–5.9)	4.7 (3.7 to 5.7)	5.4 (4.3 to 6.5)	5.0 (4.2 to 5.8)	2% increase
**EVI, PVA <6/12 to 6/18**	NA	NA	NA	7.3 (6.1 to 8.4)	7.9 (6.4 to 9.4)	7.6 (6.6 to 8.5)	

Extrapolating these estimates to the country’s population, among the people aged ≥ 50 years (115,178) in 2018, there are 16,335 (14.2%) visally impaired people at age 50 or above that includes 1,151 blind, 691 SVI, 5,759 MVI, and 8,734 EVI people.

### Causes of blindness and visual impairment

In 2018, the leading causes of visual impairment including blindness were cataract (48.4%) uncorrected refractive error (URE) (26.0%), posterior segment diseases (15.2%), glaucoma and corneal lesions (2.6% each). The major causes of bilateral blindness were cataract (53.8%), glaucoma (12.3%), non-trachomatous corneal opacity (9.2%) and posterior segment disorders (7.7%). (Table 2) The study showed that 88.9% of visual impairment (all forms) were avoidable (treatable or preventable). The comparison of the causes of blindness, and visual impairment between two RAAB studies in 2009 and 2018 is shown in **Table 2.**

**Table 2:** Comparison of overall degree and causes of blindness, SVI, MVI and EVI.

Sl.No:	Cause	RAAB 2009 (%)					RAAB 2018 (%)				
		Blindness	SVI	MVI	EVI	All	Blindness	SVI	MVI	EVI	All
1	Refractive error	0	5.6	34.7	NA	**23.2**	1.5	7.1	5	46.7	**25.9**
2	Aphakia uncorrected	1.5	1.9	0.5	NA	**0.8**	0	0	0	0	**0**
3	Cataract untreated	67.6	74.1	57.1	NA	**61.9**	53.8	57.1	65.3	34.3	**48.3**
4	Cataract surgical complications	1.5	1.9	1.4	NA	**1.4**	3.1	7.1	5.6	4.1	**4.7**
5	Pterygium	-	-	-	NA	**-**	1.5	4.8	0	4.6	**2.6**
6	Corneal opacity (Non Trachoma	1.5	1.9	0.5	NA	**0.8**	9.2	4.8	2.6	1.5	**2.6**
7	Phthisis	5.9	0	0	NA	**1.1**	4.6	0	0	0	**0.3**
8	Myopic Degeneration	-	-	-	NA	**-**	0	2.4	0	0.2	**0.2**
9	Glaucoma	5.9	0	0.9	NA	**1.7**	12.3	0	3.5	0.9	**2.6**
10	Diabetic retinopathy	0	0	0.5	NA	**0.3**	1.5	0	2.9	0.7	**1.5**
11	ARMD	1.5	7.4	0.9	NA	**2.1**	3.1	4.8	5.9	4.6	**4.9**
12	Other posterior segment disease/CNS abnormalities	14.7	5.6	3.2	NA	**5.8**	9.2	11.9	9.1	2.4	**5.2**
**Comparison by intervention categories (in person)**											
A	Treatable (#1,2,3)	69.1	81.5	92.2	NA	**85.9**	55.4	64.3	70.3	81	**74.3**
B	Preventable (PHC/PEC services) (#6,7)	8.8	3.7	1.8	NA	**3.5**	15.4	11.9	2.7	6.3	**3.0**
C	Preventable (Ophthalmic services) (#4,5,9,10)	5.9	0	1.4	NA	**3.5**	16.9	7.1	12.1	5.7	**11.6**
D	Avoidable (A+B+C)	83.8	85.2	95.4	NA	**92.9**	87.7	83.3	85	93	**88.9**
E	Posterior Segment Causes (#8,9,10,11,12)	22.1	14.8	5.9	NA	**10.6**	24.6	19.1	20.3	8.5	**15.2**

EVI- Early Visual Impairment; MVI- Moderate Visual Impairment; SVI- Severe Visual Impairment

### Cataract and visual impairment

In the sample population, 0.6% (n = 28) people were bilaterally blind due to cataract. Adjusting for age and gender, the prevalence of cataract that caused visual impairment (all categories) in person was 8.3%. The comparison of the adjusted results for prevalence of cataract in 2009 and current RAAB is shown in Table 3.

**Table 3:** Comparison of adjusted results for cataract and visual acuity with best correction.

BCVA or pinhole	Laterality	2009			2018		
		Male	Female	Total	Male	Female	Total
		% (n)	% (n)	% (n)	% (n)	% (n)	% (n)
Cataract & VA < 3/60	Bilateral cataract	0.6 (276)	0.9 (388)	0.7 (663)	0.2 (111)	0.6 (355)	0.4 (466)
	Unilateral cataract	2.6 (1,246)	3.4 (1,466)	3.0 (2,713)	1.0 (626)	1.5 (824)	1.3 (1,450)
	Cataract eyes	1.9 (1,798)	2.6 (2,241)	2.2 (4,039)	1.1 (1,272)	1.6 (1,798)	1.3 (3,070)
Cataract & VA < 6/60	Bilateral cataract	0.52 (249)	1.02 (439)	0.76 (688)	0.3 (182)	0.8 (422)	0.5 (604)
	Unilateral cataract	1.17 (535)	1.13 (486)	1.13 (1,021)	1.3 (803)	1.9 (1,050)	1.6 (1,853)
	Cataract eyes	1.07 (1,014)	1.38 (1,185)	1.22 (2,199)	1.4 (1,686)	2.1 (2,311)	1.7 (3,997)
Cataract & VA < 6/18	Bilateral cataract	1.98 (937)	1.02 (439)	0.76 (668)	2.2 (1,322)	2.6 (1,402)	2.4 (2,724)
	Unilateral cataract	2.58 (1,224)	1.13 (486)	1.13 (1,021)	2.7 (1,620)	4.0 (2,179)	3.3 (3,799)
	Cataract eyes	3.0 (2,838)	1.07 (1,185)	1.22 (2,199)	4.0 (4,781)	5.2 (5,679)	4.5 (1,0460)

BCVA- Best Corrected visual Acuity

### Cataract services in Bhutan

Most of the people underwent cataract surgery with intraocular lens (IOL) implantation [97.6%]. The CSC of eyes has increased in last 8 years and the eCSC was available for the first time in 2018 survey (Table 4). The eCSC was 67.3% for blind people (VA< 3/60). It was consistently better in urban than rural population though, the difference was not statistically significant (all P>0.05) S2 Table.

**Table 4:** Cataract surgical coverage (CSC) and Effective cataract surgical coverage (eCSC) (persons).

Category	VA	RAAB 2009			RAAB 2018		
		Male %	Female %	Total %	Male %	Female %	Total %
CSC	< 3/60	77.8	67.7	72.7	91.3	82.8	86.1
	< 6/60	67.1	51.1	58.6	86.4	81.1	83.2
	< 6/18	46.3	33.3	39.4	51.4	59.1	55.6
eCSC	< 3/60	NE	NE	NE	68.8	66.4	67.3
	< 6/60	NE	NE	NE	62.5	62.9	62.7
	< 6/18	NE	NE	NE	33.9	44.2	39.5

NE- not estimated; RAAB-Rapid Assessment of Avoidable Blindness

### Visual outcome after cataract surgery

**Fig 1:**
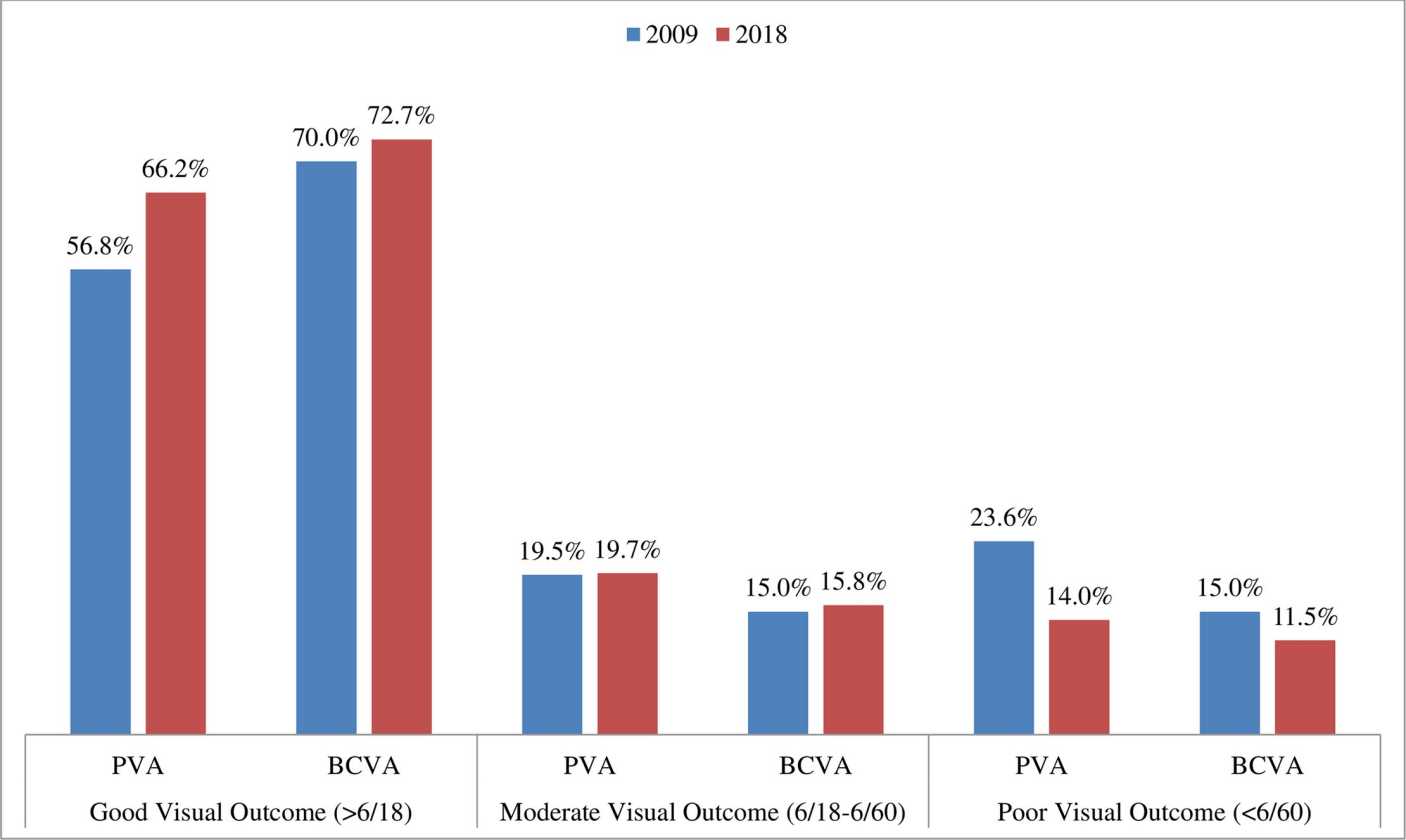
The comparision of cataract surgery visual outcome between 2009 and 2018.

Good post operative visual acuity post cataract surgery (> 6/18) was achieved in 66.2% eyes for the country, and in 14% eyes it was < 6/60. A relative improvement of cataract surgical visual outcome from 2009 and 2018 is shown in Fig 1.

The Effective Cataract Surgical Coverage (eCSC), was 67.3% for blindness (VA< 3/60). Though the eCSC was consistently better in urban than rural population, the difference was not statistically significant (all P>0.05). **(Table 5).**

**Table 5:** Visual outcome (PVA) after cataract surgery in 2018.

Visual Outcome		Very good: can see 6/12	Good: can see 6/18	Borderline: can see 6/60	Poor: cannot see 6/60
**Type of Surgery**	**Non-IOL**	0.0	7.7	30.8	61.5
	**IOL**	50.6	17.2	19.5	12.8
**Place of surgery**	**Government Hospital**	48.8	16.9	19.8	14.5
	**Abroad**	40.9	9.1	18.2	31.8
	**National Eye camp**	52.9	19.0	18.3	9.8
	**International Eye camp**	41.7	12.5	29.2	16.7
**Years after surgery**	**3 years**	52.3	17.2	18.4	12.1
	**4–6 years**	57.0	14.1	19.5	9.4
	**7+ years**	39.4	18.8	21.8	20.0
**Causes**	**Co-morbidity**	0.0	16.7	39.7	43.6
	**Surgery**	0.0	21.6	37.8	40.5
	**Spectacle**	0.0	75.0	25.0	0.0
	**Long term sequelae**	0.0	34.2	43.6	22.2

The visual outcome was better in the IOL implanted eyes (72.7% vs 23.1%). The locations of cataract surgeries were Government Hospitals (62.9%), National Eye Camps (28.5%), International Eye Camps (4.5%) and outside of the country (4.1%). The main cause of poor outcome after cataract surgery were co-morbidities (43.6%), surgery-related (40.5%) and long term sequelae (22.2%) **[Table 5].** Co-morbidities included corneal opacities and other posterior segment diseases.

### Barriers to cataract surgery

A total of 56 cataract blind (un-operated bilateral cataract and best corrected visual acuity, BCVA, < 6/60) participants were interviewed to identify reasons for not availing cataract surgery. The significant barriers were the ‘lack of accompanying person’ and ‘distance’ (37.5%), ‘ignorance of available treatment’ (19.6%) and ‘need not felt’ (19.6%). No one reported financial reason as a barrier. The gender specific barriers were the ‘lack of accompanying person’ (41.7%) in women and ‘need not felt’ (35.0%) in men. The location specific barriers were the ‘lack of accompanying person’ (35.4%) for rural population and ‘need not felt’ (37.5%) for urban population.

## Discussion

The prevalence of blindness in the South East Asia Region (SEAR) ranges between 0.6% to 8%. [14–20] The current survey estimated the prevalence of blindness in Bhutan at 1.0%, a decrease by 33% from 1.5% estimated in 2009 RAAB survey. The relatively lower prevalence of bilateral blindness in Bhutan could be attributed to the integration of eye care with the general health care program that provides free basic health care to the citizen through a three tier health care delivery system and a strong political commitment. Furthermore, the continued financial and technical support provided by international agencies and donors (IAPB, Himalayan Cataract Project, HCP) and a well established Primary Eye Care Program (PECP) to coordinate, implement and monitor eye care services have aided the eye care services in Bhutan.

Women accounted for 68.4% of blindness (male 1%, female 1.6%) and 75% of cataract blindness (men 0.2%, women 0.6%) in Bhutan. This is similar to other reports. [21,22]. Women of all ages tend to utilise eye care services less than men and this results in more blindness and visual impairment in them. [23] A relatively longer life expectancy for women (71.7 years) than men (68.8 years) in Bhutanese population could be an additional factor and this will be an important consideration in future.

The majority (88.9%) of the blindness and visual impairment in this study were avoidable. Cataract and refractive error constituted 55.3% of blindness and 74% of visual impairment. The leading causes of all visual impairment were cataract (48.4%), uncorrected refractive error (25.9%) and posterior segment diseases (15.4%). This was consistent with the WHO reported global data in 2015. [24]

The number of people with cataract and refractive error is expected to increase. Strengthening of the eye care services with ophthalmologists and allied ophthalmic personnel (AOP) should help reduce avaoidabe blindness and visual impairment. One ophthalmologist and one AOP for every 100,000 people are recommended. Based on the country population, Bhutan needs 22 ophthalmologoists and 43 AOPs. [25] The current available numbers are inadequate. [26] In the current survey posterior segment diseases such as glaucoma, diabetic retinopathy and age-related macular degeneration caused 15.4% of all visual impairment and 26.1% of blindness. A higher resource investment is needed for care of these conditions.

The cataract services in Bhutan with the cataract surgical coverage at 86.1% meets the WHO target of 85% and reflects the success of national eye care programs. While 94.3% people received an IOL, good postoperative vision (VA ≥ 6/18) at 67.3% was below the WHO recommendation of 85%. The reasons were coexisting ocular comorbidity (43.6%, similar to other studies [27,28] and surgical complications including postoperative sequelae (62.7%). This calls for the improvement in the quality of cataract surgery.

Extrapolating the RAAB 2018 estimates for the country’s population, 0.4% (n = 466) people are blind, 0.5% (n = 604) people have SVI, 2.45% (n = 2,247) people have MVI and 5.0% (n = 5,755) people have EVI due to cataract. The current cataract surgical rate in Bhutan at 1550/million people is far behind India, Nepal, and Sri Lanka. [26] Despite good CSC, the number of people needing surgery for blinding/ visually impairing cataract remains high in Bhutan. With improvement in cataract surgical technique, operating cataract at visually impaired state (VA <6/18) than the blinding state (VA <3/60) is more often considered. [29,30]. If the cutoff for cataract surgery is set to 6/18, then 10,460 people would need cataract surgery in Bhutan. This additional need can be explained in two ways: 1) the coverage failed to reach a significant number of people with blinding cataract, 2) the cataract surgical rate is not high enough to absorb new cases of blinding cataract. [31] New approaches are required to address this burden of cataract. The prominent barriers among unoperated cataract blind people were accessibility and lack of an accompanying attendant. This could be improved if the cataract surgical services could be taken closer to people. The integration of eye care with the health care program to provide free health services as well as the financial support of the external donors have served its purpose in a major way. But the indirect cost such as travel cost and wages loss of accompanying person have not reduced.

### The changing trends in eye care services

**Fig 2:**
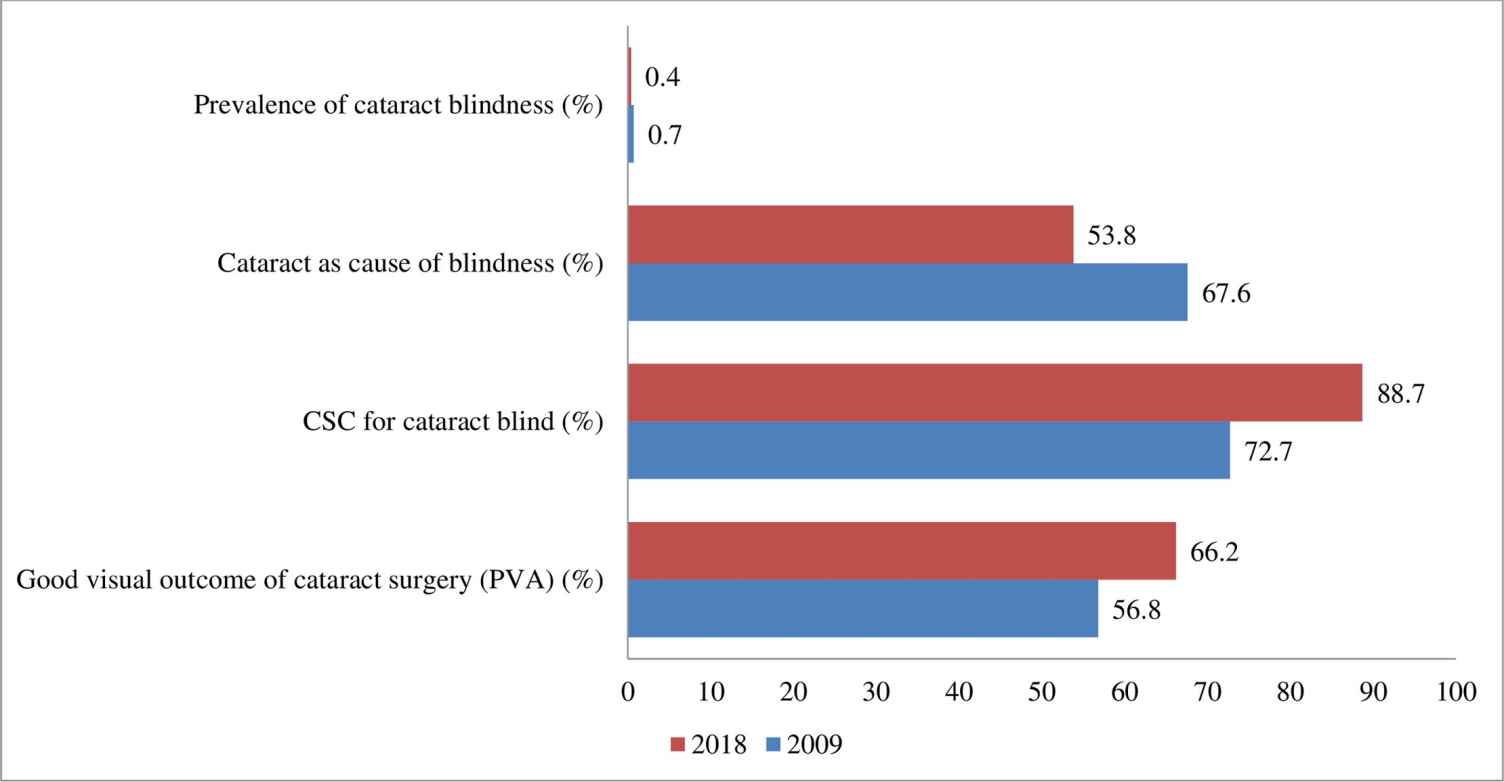
Cataract service indicators to measure progress from 2009 to 2018.

The prevalence of blindness and SVI has decreased from 2009 and this reduction is encouraging, so also the significant reduction in the prevalence of cataract blindness (0.7% in 2009 and 0.4% in 2018) (Fig 2) But the population growth and ageing contribute to a substantial increase in the number of blind or visually impaired people. [8] Added to this, is the increasing prevalence of posterior segment diseases. These findings warrant accelerating cataract surgical services, augmenting refraction services and strengthening retinal services in Bhutan.

The strength of the study was its nationwide inclusion with the population cohort pooled from all 20 districts of the country, a systematic examination of the recruits using the accepted standards RAAB, a high response rate (98.4%) and a good interobserver agreement. Training by certified RAAB trainers, experienced principal investigator and enumerator, and regular monitoring of field work by survey coordinators ensured a high quality data acquisition. We acknowlege that this survey was not an all-age-group study and the known limitations of RAAB applies to the results. One such important limitation is the fact that blindness is defined by visual acuity rather than visual function.

## Conclusion

Bhutan has achieved the target of WHO Universal Eye Health Global Action Plan 2014–2019 in the blindness category with a decrease in prevalence of blindness by 33% from its baseline survey of 2009. This study provides evidence to show that Bhutan is on track to achieving the goals of VISION 2020. This achievement could be attributed to increased eye health human resource, infrastructure and eye health activities in the country.

However, other categories of visual impairment are high. The avoidable causes (cataract and refractive error) are the major burden of diseases and gender discrepancies still persist in eye care services of Bhutan. This calls for a need not only to sustain the ongoing program but to further develop and deploy human resources, accelerate cataract surgical services, augment refraction services and strengthen retinal services. To effectively and sustainably address the challenges, Bhutan should continue to implement long-term strategic action plan for eye health focused on strengthening primary eye care and comprehensive eye care services.

## Supporting information

S1 Table Age and gender distribution of the sample and the census population, 2018. (DOCX)

S2 Table Bhutan Cataract Surgical Coverage (CSC) in 2018. (DOCX)

S1 File Bhutan RAAB raw data. (XLS)
