# Supplementary material for: Changing trends of blindness, visual impairment and cataract surgery in Bhutan: 2009–2018
Source: PLoS One. 2019 May 9;14(5):e0216398. doi: 10.1371/journal.pone.0216398 (PMC6508732; doi:10.1371/journal.pone.0216398)
Supplement: S1 Table — (DOCX) [file pone.0216398.s001.docx]

**Supporting Information**

**S1 Table: Age and gender distribution of the sample and the census population, 2018**

| **Age Group** | **Male** | | **Female** | | **Total** | |
| --- | --- | --- | --- | --- | --- | --- |
|  | **Sample**  **n (%)** | **Census**  **n (%)** | **Sample**  **n (%)** | **Census**  **n (%)** | **Sample**  **n (%)** | **Census**  **n (% )** |
| **50-59** | 831 (34.7) | 3,0106 (49.8) | 979 (38.1) | 26,268 (48.9) | 1,810 (36.4) | 56,374 (48.9) |
| **60-69** | 776 (32.4) | 18,291 (30.3) | 841 (32.7) | 16,621 (30.4) | 1,617 (32.5) | 34,912 (30.3) |
| **70-79** | 504 (21.0) | 8,675 (14.4) | 531 (20.6) | 8,517 (15.6) | 1,035 (20.8) | 17,192 (14.9) |
| **80+** | 287 (12.0) | 3,356 (5.6) | 221 (8.6) | 3,344 (6.1) | 508 (10.2) | 6,189 (5.8) |
| **Total** | 2,398 (100) | 60,428 (100) | 2,472 (100) | 54,750 (100) | 4,970 (100) | 115,178 (100) |
