# Supplementary material for: Changing trends of blindness, visual impairment and cataract surgery in Bhutan: 2009–2018
Source: PLoS One. 2019 May 9;14(5):e0216398. doi: 10.1371/journal.pone.0216398 (PMC6508732; doi:10.1371/journal.pone.0216398)
Supplement: S2 Table — (DOCX) [file pone.0216398.s002.docx]

**Supporting Information**

**S2 Table:Bhutan Cataract Surgical Coverage (CSC) in 2018**

|  | **Rural** | | | **Urban** | | | **Total** | | |
| --- | --- | --- | --- | --- | --- | --- | --- | --- | --- |
|  | **Males** | **Females** | **Total** | **Males** | **Females** | **Total** | **Males** | **Females** | **Total** |
| **Cataract Surgical Coverage (eyes)- %** | | | | | | | | | |
| VA < 3/60 | 74.3 | 70.9 | 72.4 | 81.1 | 77.6 | 79.2 | 76.7 | 73.1 | 74.7 |
| VA < 6/60 | 68.6 | 64.6 | 66.3 | 76.3 | 75.4 | 75.8 | 71.3 | 68.1 | 69.5 |
| VA < 6/18 | 43.1 | 42.6 | 42.8 | 50.9 | 56.2 | 53.6 | 45.7 | 47.6 | 46.2 |
| **Cataract Surgical Coverage (persons) – %** | | | | | | | | | |
| VA < 3/60 | 90.6 | 78.8 | 83.5 | 92.6 | 90.5 | 91.3 | 91.3 | 82.8 | 86.1 |
| VA < 6/60 | 83.1 | 76.7 | 79.2 | 93.1 | 90.5 | 91.5 | 86.4 | 81.1 | 83.2 |
| VA < 6/18 | 49.2 | 53.3 | 51.5 | 56.4 | 75.0 | 65.8 | 51.4 | 59.1 | 55.6 |
| **Effective Cataract Surgical Coverage (persons) – %** | | | | | | | | | |
| VA < 3/60 | 69.8 | 60.0 | 63.9 | 66.7 | 78.6 | 73.9 | 68.8 | 66.4 | 67.3 |
| VA < 6/60 | 62.7 | 55.6 | 58.4 | 62.1 | 78.6 | 71.8 | 62.5 | 62.9 | 62.7 |
| VA < 6/18 | 33.6 | 37.5 | 35.8 | 34.6 | 62.5 | 48.7 | 33.9 | 44.2 | 39.5 |
